# An examination of factorial invariance of the Asthma Control Questionnaire among adults with severe asthma

**DOI:** 10.1371/journal.pone.0295493

**Published:** 2023-12-07

**Authors:** Ronald McDowell, Liam Heaney, Thomas Brown, Brendan Bunting, Hassan Burhan, Rekha Chaudhuri, Paddy Dennison, Shoaib Faruqi, Robin Gore, David J. Jackson, Andrew Menzies-Gow, Thomas Pantin, Mitesh Patel, Paul Pfeffer, Salman Siddiqui, John Busby, on behalf of the UK Severe Asthma Registry

**Affiliations:** 1 School of Medicine, Dentistry and Biomedical Sciences, Queen’s University, Belfast, United Kingdom; 2 School of Psychology, Ulster University, Coleraine, United Kingdom; 3 Belfast Health & Social Care NHS Trust, Belfast, United Kingdom; 4 Portsmouth Hospitals University NHS Trust, Portsmouth, United Kingdom; 5 Royal Liverpool Hospital, Liverpool, United Kingdom; 6 Gartnavel General Hospital, Glasgow, United Kingdom; 7 University Hospital Southampton NHS Foundation Trust, Southampton, United Kingdom; 8 Hull University Teaching Hospitals NHS Trust, Hull, United Kingdom; 9 Cambridge University Hospitals NHS Foundation Trust, Cambridge, United Kingdom; 10 Guy’s Severe Asthma Centre, Guy’s and St Thomas’ Hospitals, London, United Kingdom; 11 School of Immunology & Microbial Sciences, King’s College London, London, United Kingdom; 12 Royal Brompton and Harefield Hospitals, London, United Kingdom; 13 Wythenshawe Hospital, Manchester NHS Foundation Trust, Manchester, United Kingdom; 14 University Hospitals Plymouth NHS Trust, Plymouth, United Kingdom; 15 Barts Health NHS Trust, London, United Kingdom; 16 National Heart and Lung Institute, Imperial College, London, United Kingdom; Girne American University - Karmi Campus: Girne Amerikan Universitesi, CYPRUS

## Abstract

**Background:**

The Asthma Control Questionnaire (ACQ) is used to assess asthma symptom control. The relationship between the questionnaire items and symptom control has not been fully studied in severe asthmatic patients, and its validity for making comparisons between subgroups of patients is unknown.

**Methods:**

Data was obtained from patients in the United Kingdom Severe Asthma Registry whose symptom control was assessed using the five-item ACQ (ACQ5) (n = 2,951). Confirmatory factor analysis determined whether a latent factor for asthma symptom control, as measured by the ACQ5, was consistent with the data. Measurement invariance was examined in relation to ethnicity, sex and age; this included testing for approximate measurement invariance using Bayesian Structural Equation Modelling (BSEM). The fitted models were used to estimate the internal consistency reliability of the ACQ5. Invariance of factor means across subgroups was assessed.

**Results:**

A one-factor construct with residual correlations for the ACQ5 was an excellent fit to the data in all subgroups (Root Mean Square Error Approximation 0.03 [90%CI 0.02,0.05], p-close fit 0.93, Comparative Fit Index 1.00, Tucker Lewis Index 1.00}. Expected item responses were consistent for Caucasian and non-Caucasian patients with the same absolute level of symptom control. There was some evidence that females and younger adults reported wakening more frequently during the night than males and older adults respectively with the same absolute level of symptom control (p<0.001). However approximate measurement invariance was tenable and any failure to observe strong measurement invariance had minimal impact when comparing mean levels of asthma symptom control between patients of different sexes or ages. Average levels of asthma symptom control were lower for non-Caucasians (p = 0.001), females (p<0.01)and increased with age (p<0.01). Reliability of the instrument was high (over 88%) in all subgroups studied.

**Conclusion:**

The ACQ5 is informative in comparing levels of symptom control between severe asthmatic patients of different ethnicities, sexes and ages. It is important that analyses are replicated in other severe asthma registries to determine whether measurement invariance is observed.

## 1. Introduction

Asthma is a chronic respiratory condition which affects an estimated 339 million people worldwide [1]. The Global Initiative for Asthma (GINA) ranks asthma severity as intermittent, mild intermittent, moderate persistent and severe persistent, dependent on symptoms, airflow limitation and lung function [2]. Patients with severe asthma (defined as “asthma which requires treatment with high dose inhaled corticosteroids plus a second controller and/or systemic corticosteroids to prevent it from becoming ‘uncontrolled’ or which remains ‘uncontrolled’ despite this therapy” [3]) account for between 5% and 10% of asthma patients, and typically suffer from significant morbidity [4], mortality [5] and poor quality-of-life [6].

Asthma control is the degree by which the manifestations of asthma are observed, or have been reduced/removed by treatment, and comprises two domains-symptom control and risk factors for future exacerbations [7]. These domains are often associated but may be discordant (patients with well-controlled symptoms may still be prone to frequent exacerbations and vice-versa), particularly among patients with severe asthma [8]. Given that asthma control cannot be quantified using a single measure [9], multiple-item instruments have been developed. These include the Asthma Control Test (ACT) [10], the Royal College of Physicians Three Questions (RCP3Q) [11] and the Asthma Control Questionnaire (ACQ) [12]. The ACQ was the first structured questionnaire designed to measure asthma control [13]. It measures asthma symptom control using seven items ranked on a seven-point ordinal scale from no impairment (0 points) to maximum impairment (6 points). The first six items are self-assessed and relate to the extent over the past week the patient reported their asthma woke them from their sleep (acq1), the severity of their symptoms when waking (acq2), limitations in activities (acq3), shortness of breath (acq4), wheezing (acq5), and daily use of short-acting bronchodilators/beta-agonists (SABAs) (acq6). The seventh item is the Forced Expiratory Volume in 1 second (FEV_1_) % predicted as measured by a clinician (acq7). A patient’s ACQ score can be reported as the average of the first five, six or seven questions depending on the availability of data and/or clinical context.

The ACQ is considered a valid and reliable instrument for assessing asthma symptom control in adults with mild/moderate asthma [13]. There are concerns that instruments designed for assessing health-related quality of life (QoL) in asthma patients may be unsuitable for severe asthmatic patients as they may fail to assess deficits specific to severe asthma [14]. Nevertheless, these instruments are commonly used in research settings and clinical trials among patients across the spectrum of asthma severity. To-date studies which examine the measurement properties of the ACQ in relation to asthma symptom control have been undertaken among children [15] and adults [16], however, no such studies have taken place in severe asthmatic adult populations. This patient group is important as it drives much of the morbidity and healthcare costs of asthma [17]. Furthermore, it is important that the measurement model of the ACQ is stable across subgroups of patients. This is known as measurement invariance and is a necessary prerequisite for determining whether average levels of asthma control differ between subgroups (factor means invariance). Non-invariance of patient-reported outcome measures (PROMs) has been observed [18] and this may lead to inaccurate diagnoses, inappropriate treatment and biased inferences drawn from other statistical analyses. Consequently the purpose of this study is to assess the measurement invariance properties of the ACQ among patients with severe asthma and to examine whether differences in factor mean levels of asthma control exist between subgroups of patients, specifically patients of different ethnicities, sex or age.

## 2. Materials and methods

### 2.1 Study subjects & data source

The UK Severe Asthma Registry (UKSAR) is the largest national registry of its kind and contains demographic, clinical and treatment characteristics of patients referred to UK specialist asthma centres [19]. Approval for collection and analysis of pseudonymised UKSAR data has been granted by Office for Research Ethics Committees Northern Ireland (ORECNI) (12/NO/0196). Data was analysed from UKSAR adults who met the European Respiratory Society American Thoracic Society (ERS/ATS) criteria for severe asthma and whose asthma symptom control was assessed using the ACQ at their initial visit to the specialist centre from 2014 onwards Patients with incomplete responses to the ACQ were retained (see 2.4), but those receiving biologic therapies at time of referral were excluded to increase the homogeneity of the cohort. These patients were most likely referred from another centre and already under the care of a severe asthma specialist. They would be expected to have substantially improved asthma control than newly-referred patients and thus their inclusion could potentially confound any results. A very small number of patients were excluded from centres with low patient numbers or poor data completeness in order to enhance data quality.

### 2.2 Statistical analysis

#### 2.2.1 Overview

For the purposes of this study factorial invariance of asthma symptom control as measured by the ACQ5 was studied at the initial presentation of patients to their local severe asthma clinic. Since 2020 the recommended treatment for patients with severe persistent asthma has been single-inhaler maintenance and reliever therapy (SMART) [20], however the six-item version of the ACQ (ACQ6) has not yet been evaluated in patients prescribed SMART. Asthma symptom control was considered a latent variable, measured by the ACQ5 using five observed variables (items acq1-acq5). A latent variable, sometimes called an unmeasured variable, a factor, an unobserved variable or a construct, is a variable which cannot be observed directly and hence cannot be measured directly [21, 22]. Consequently it is measured indirectly using multiple measurable items. Examples of latent variables include depression [23], disability [24] and quality of life [25]. Three sets of analyses were conducted assessing measurement invariance across ethnicity, sex (male, female) and age (18–34,35–54, ≥55 years). Ethnicity was recorded according to Global Lung Initiative (GLI) criteria (Caucasian, South-East Asian, North-East Asian, African, Mixed and Other), however, due to low numbers of patients within some groups the primary ethnicity invariance analysis compared Caucasian with non-Caucasian patients.

#### 2.2.2 Assessment of factorial invariance

Assessment of factorial invariance comprises two key components, testing for measurement invariance and testing for structural invariance. Attainment of measurement invariance is usually considered a necessary prerequisite before making substantive cross-group comparisons [26]. The assessment of measurement invariance is a hierarchical process and is summarised below.

**Fig 1:**
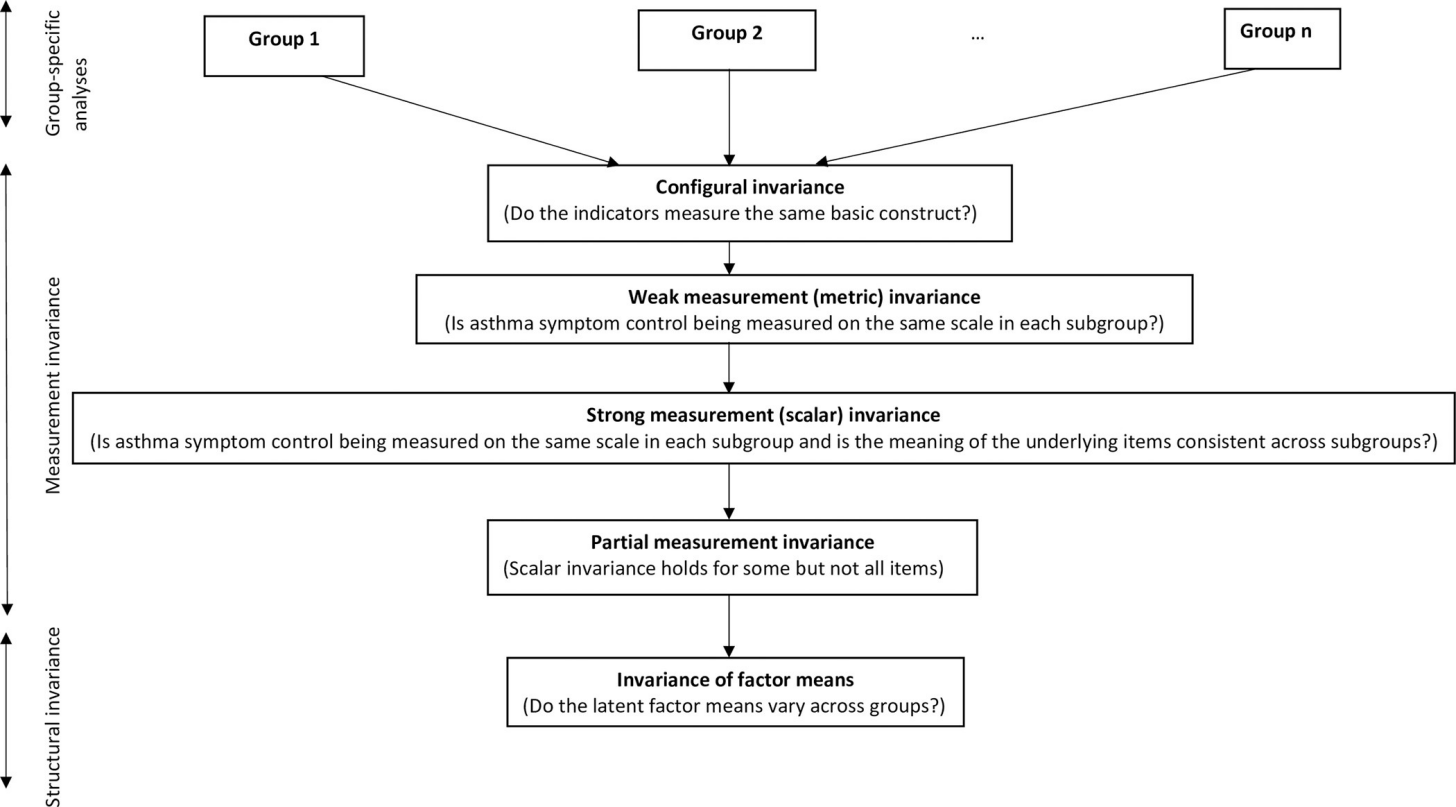
Steps in the assessment of factorial invariance of the Asthma Control Questionnaire.

**Fig 2:**
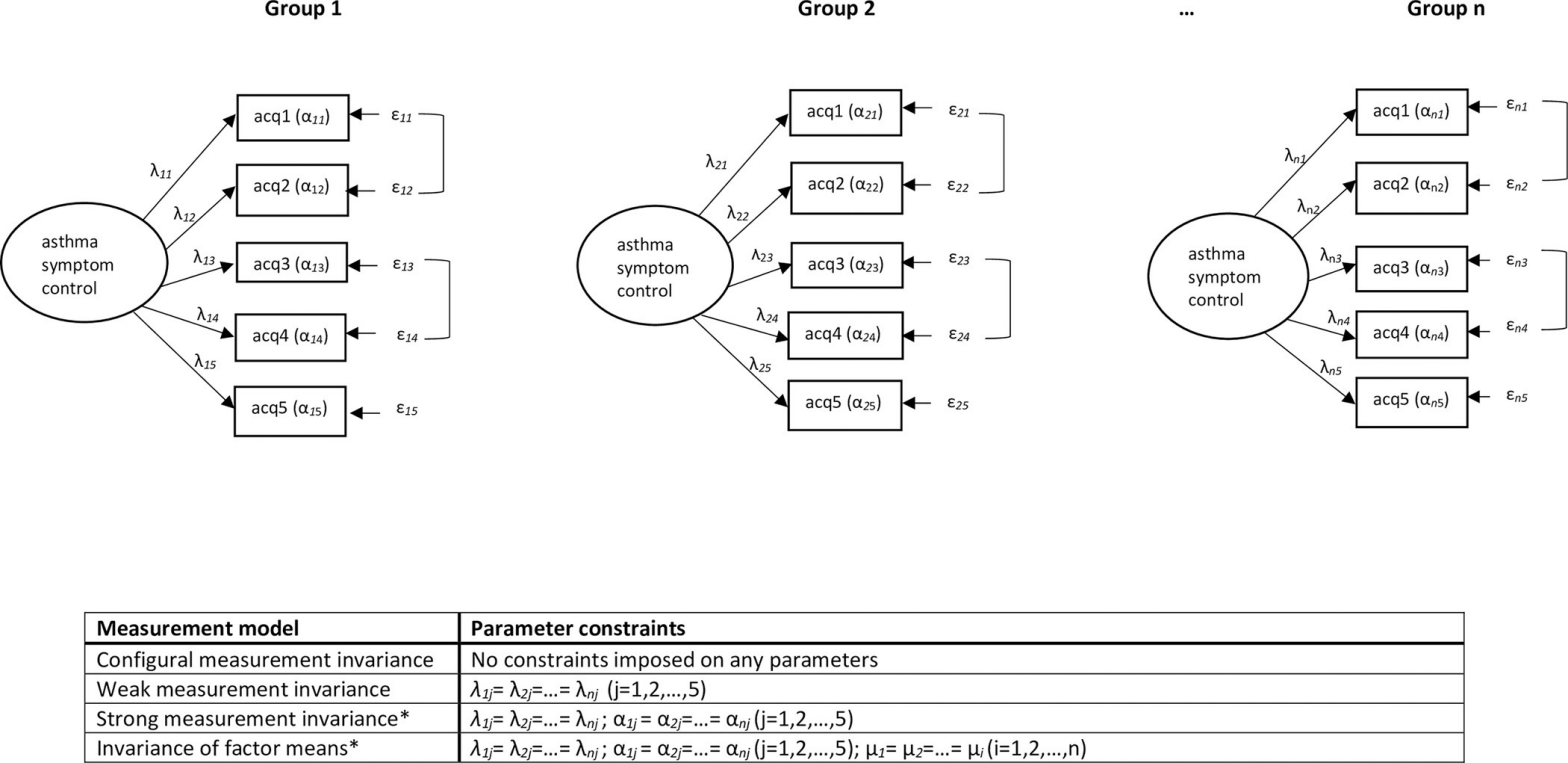
Asthma control as measured by the ACQ5. For group i = 1,2,…,n and item j = 1,…,5, each item acqj has a loading (λ_*ij*_), an intercept (α_*ij*_) and an error term (ε_*ij*_). Each group has its own latent factor with mean (μ_*i*_) and variance (σ^2^_*i*_). *Partial measurement invariance may hold when constraints imposed on the intercepts (α_*ij*_) are relaxed for one or more items in any subgroup.

Confirmatory factor analysis (CFA) was used to determine whether the proposed relationship between asthma symptom control and the items of the ACQ5 was observed in each subgroup. Where lack-of-fit was observed i.e. the model was considered unable to reproduce the data, usually the variance/covariance matrix [27], consideration was given to whether model fit would be improved through permitted modifications which were considered clinically relevant. This included he inclusion of correlated residuals between questionnaire items (indicating a local dependency between items above that implied by the model and whose omission have the potential to bias the other parameters) [21, 27]. For each analysis in turn (ethnicity, sex, age) individual subgroup models were pooled and a sequence of nested models fitted, ranging from the weakest to the strongest form of measurement invariance. These are described in Figs 1 and 2. The potential for partial measurement invariance was considered where strong measurement invariance failed for some items [28]. Where this was observed a test of approximate measurement invariance was applied using Bayesian Structural Equation Modelling (BSEM). This is an enhanced version of conventional Structural Equation Modelling (SEM) which uses small-variance priors to build more flexible and realistic sets of models, as the criteria commonly used to assess strong measurement invariance may be overly strict for practical purposes [29].

Having assessed measurement invariance tests of factor means invariance were undertaken. Finally, estimated factor means were compared with mean ACQ5 summary scores; these were calculated as the average response to the ACQ5 questionnaire items for each patient. This comparison was used to determine whether observed summary scores, calculated assuming measurement invariance, mirror unobserved factor scores where measurement invariance has been assessed, and hence can be used in clinical practice to inform whether differences in asthma control exist between subgroups of patients.

Within each subgroup the final measurement model was used to estimate the internal consistency reliability of the ACQ5. This approach is considered preferable to other methods as it allows for a test of the underlying model used to describe the data [30].

#### 2.2.3 Assessment of model fit

Fit statistics were examined to determine the extent to which the hypothesised models and data were considered in agreement. These included absolute fit indices e.g. the Root Mean Square Error of Approximation (RMSEA), test of close fit, Standardised Root Mean Square Residual (SRMR) and relative fit indices e.g. Comparative Fit Index (CFI), Tucker Lewis Index (TLI)., Values of RMSEA <0.5, SRMR<0.8, test of close fit p>0.05, CFI >0.95, TLI >0.95 were considered to indicate good fit and lack of evidence to reject the model [31, 32]. The chi-square statistic was not used to inform model choice due to its well-documented sensitivity to sample size [33]. Nested models were compared using the Information Criterion (particularly the Bayesian Information Criterion (BIC) and Sample Size Adjusted BIC (SSA-BIC)), and changes in the CFI (a change <0.1 was considered acceptable) [34]. Univariate modification indices (MI), the expected change in the log-likelihood if the parameter associated with the MI was introduced into the model [35], were examined for evidence of possible model misspecification.

### 2.3 Sensitivity analyses

The ethnicity analysis was rerun using all six ethnicity groups. Patients were split using an alternative categorisation for age (18–45, >45 years); the probability of severe asthma increases at a lower rate after 45 years [36]. Age was also treated as a continuous variable, with measurement invariance assessed using a Multiple Indicator Multiple Causes (MIMIC) model. The one factor structure for the ACQ5 was retained but age was considered as an observed predictor which affects or “causes” the latent factor of asthma symptom control. The main analyses were rerun allowing for clustering of patients within sites. An additional sensitivity analysis was undertaken comparing patients who were Type-2 biomarker high with other patients. These patients have high blood eosinophil counts (≥0.15 10^9^/L) and high levels of Fractional Exhaled Nitric Oxide (FeNO) (> = 0.25 ppb)), both of which are markers of inflammation of the airways. Consequently patients who are Type-2 biomarker high are more likely to have uncontrolled asthma and are at increased risk of exacerbations [37]. The UKSAR has been shown to have higher numbers of Type-2 biomarker high patients compared to other severe asthma registries [38]. Finally, the main analyses were repeated using the ACQ6. The ACQ6 is an extended version of the ACQ5 which includes an additional item (question 6) detailing a patient’s self-reported daily use of SABAs, rated from 0 points (none) to 6 points (more than 16 puffs a day). SABAs are intended for short-term relief of asthma and hence overreliance may indicate poor asthma control. The ACQ6 continues to be commonly reported in the severe asthma literature.

### 2.4 Use of statistical software

Descriptive statistics were calculated using Stata 16 SE [39], with the factor analyses undertaken using Mplus 7.3 [40]. In the absence of absolute measurement scales for latent variables, the intercept for the first item (acq1) was initially fixed at zero in each group (marker variable approach). This approach allowed us to freely estimate the factor means for asthma control in all subgroups and is mathematically equivalent to analyses where the factor mean is fixed at zero in one subgroup and other factor means are estimated relative to this reference group. All models were estimated using the Maximum Likelihood Robust (MLR) estimator, which is robust against deviations from normality [41], and used all available data under the missing at random (MAR) assumption.

## 3. Results

### 3.1 Descriptive statistics

Table 1 details the demographic and clinical characteristics of the cohort (n = 2,951). Four-fifths of patients were Caucasian (81.1%, n = 2,369) and the majority were female (61.7%, n = 1,821). Median age was 52 years (Inter-Quartile Range (IQR) 41, 61), with more than half of participants diagnosed with asthma as adults (55.1%, n = 1,467). Patients on average were slightly obese (mean Body Mass Index (BMI) 30.8 (standard deviation (SD) 7.2)) and two-thirds reported never smoking (65.9%, n = 1,916).

**Table 1:** Characteristics of the study population.

Variable	N = 2,951
**Demographic variables**	
**Sex**	
Female	1,821 (61.7%)
Male	1,130 (38.3%)
**Age at presentation (Years)**	
18–34	511 (17.3%)
35–54	1,167 (39.5%)
≥55	1,273 (43.1%)
**Age of onset (Years)**	
<12	927 (34.8%)
12–18	270 (10.1%)
>18	1,467 (55.1%)
**Ethnicity**	
Caucasian	2,369 (81.1%)
South-East Asian	126 (4.3%)
North-East Asian	67 (2.3%)
African	95 (3.3%)
Mixed	24 (0.8%)
Other	240 (8.2%)
**Smoking status**	
Never	1,916 (65.9%)
Ex-smoker	859 (29.5%)
Current smoker	132 (4.5%)
**BMI (kg-m2)**	30.8 (7.2)
**Clinical measures**	
**Clinic FEV**_**1**_ **(% Predicted)**	67.0 (21.0)
**Clinic FVC (% Predicted)**	83.7 (19.1)
**Clinic FEV**_**1**_ **/FVC**	64.7 (17.1)
**Blood eosinophil count (10**^**9**^**/L)** *	0.38 (0.20,0.60)
**Highest blood eosinophil count**^**†**^ **(10**^**9**^**/L)** *	0.64 (0.40,1.00)
**FeNO (ppb)** *	40 (21,73)
**Total IgE (IU/mL)** *	150 (52,421)
**Medication and service use**	
**On maintenance oral corticosteroids**	1,444 (49.2%)
**Exacerbations requiring rescue steroids in previous year** *	4 (2,7)
**Invasive ventilations (ever)**	275 (9.9%)
**Emergency department visit/hospital admission in previous year**	1,377 (48.6%)

Categorical variables summarised as counts (%); scalar measures reported as mean (standard deviation) unless otherwise indicated

* median, inter-quartile range reported due to skewed distribution of scalar measure

^†^ as recorded in medical records

BMI: Body Mass Index; FeNO: Fractional Exhaled Nitric Oxide; FEV_1_: Forced Expiratory Volume in one second; FVC: Forced Vital Capacity; IgE: Immunoglobulin E

Poor asthma control was observed in this cohort with a median 4 (IQR 2,7) exacerbations in the year before assessment. There was significant airflow obstruction (mean predicted FEV_1_ 67.0% [SD 21.0]), with high levels of type-2 biomarkers including blood eosinophils (median 0.38 cell x 10^9^/L, IQR: 0.20,0.60) and fractional exhaled nitric oxide (FeNO) (median 40 ppb, IQR: 21, 73). Almost half of patients were on maintenance oral corticosteroids (49.2%, n = 1,444). Demographic and clinical characteristics by sex, ethnicity and age are listed in Supplementary Materials S1-S3 Tables in S1 File.

Reponses to each of the ACQ5 items were recorded for over 99% of all patients. For each item, on average, Caucasians reported lower levels of impairment than non-Caucasians (p≤0.024), and males reported lower levels of impairment than females (p<0.001). Mean item responses declined across age-groups (p<0.001, all items) (Table 2).

**Table 2:** Mean (SD) ACQ5 item scores by sex, ethnicity and age.

		Ethnicity			Sex			Age			
ACQ6 item	All patients	Caucasian (n = 2,369)	Non-Caucasian (n = 552)	p-value	Female (n = 1,821)	Male (n = 1,130)	p-value	18–34 (n = 511)	35–54 (n = 1,167)	≥55 years (n = 1,273)	p-value
**acq1 (wakening during the night due to asthma)**	2.5 (1.7)	2.5 (1.7)	2.7 (1.7)	0.002	2.8 (1.7)	2.1 (1.7)	<0.001	3.1 (1.7)	2.7 (1.7)	2.1 (1.6)	<0.001
**acq2 (severity of morning symptoms)**	2.9 (1.5)	2.8 (1.4)	3.1 (1.6)	0.002	3.0 (1.4)	2.6 (1.5)	<0.001	3.2 (1.4)	3.0 (1.5)	2.6 (1.4)	<0.001
**acq3 (limitation in activities)**	2.9 (1.5)	2.9 (1.5)	3.1 (1.6)	0.003	3.1 (1.5)	2.7 (1.6)	<0.001	3.1 (1.5)	3.0 (1.5)	2.7 (1.5)	<0.001
**acq4 (shortness of breath)**	3.4 (1.5)	3.3 (1.5)	3.5 (1.5)	0.024	3.5 (1.5)	3.1 (1.6)	<0.001	3.6 (1.5)	3.5 (1.5)	3.2 (1.6)	<0.001
**acq5 (wheezing)**	3.0 (1.6)	2.9 (1.7)	3.2 (1.6)	0.001	3.1 (1.6)	2.8 (1.7)	<0.001	3.2 (1.6)	3.2 (1.6)	2.8 (1.6)	<0.001

ACQ: Asthma Control Questionnaire; SD: standard deviation

### 3.2 Preliminary analysis

A one-factor model for asthma symptom control measured by five items (acq1-acq5) was fit to the entire cohort. This factor accounted for 76.6% of the variability in observed responses to the items. Although some fit statistics were acceptable (SRMR 0.02, CFI 0.97), others indicated poor model fit (RMSEA 0.13 [90%CI 0.11,0.14], p-close fit <0.01, TLI 0.93). Inspection of the modification indices suggested the correlations between some items were greater than those implied by the underlying factor. These were acq3 (limitation in activities) with acq4 (shortness of breath) (two day-time symptoms), and acq1 (wakening during the night because of asthma) with acq2 (severity of asthma symptoms on wakening in the morning) (two questions relating to waking/sleeping). Incorporation of these residual correlations resulted in excellent model fit (RMSEA 0.03 [90%CI 0.02,0.05]), p-close fit 0.93, CFI 1.00, TLI 1.00, SRMR <0.01). This model is reported in Supplementary Materials S4 Table in S1 File.

### 3.3 Multi-group analyses

#### 3.3.1 Subgroup analyses

Results from each subgroup analysis (ethnicity: Caucasian, non-Caucasian; sex: male, female; age: 18–34, 35–54, ≥55 years) mirrored the analysis of the entire cohort, with a one-factor model for the ACQ5 considered a highly satisfactory fit to the data following the incorporation of the above residual correlations. Standardised factor loadings for all items were greater than 0.75 (p<0.01) in each subgroup.

#### 3.3.2 Configural and weak measurement invariance

Model fitting steps are detailed in Tables 3 and 4. For each analysis in turn (ethnicity, sex, age), the hypotheses that the same basic construct was being measured in each subgroup of patients and was being measured on the same scale were consistent with the data.

**Table 3:** Model fit statistics for tests of factorial invariance of the ACQ5 (ethnicity, sex).

Ethnicity (Caucasian, non-Caucasian)										
Model	No free parameters	Chi-square (df), p-value	AIC	BIC	SSA-BIC	RMSEA (90%CI)	p-close fit	CFI (ΔCFI‡)	TLI	SRMR
**1.Configural invariance**	34	11.647 (6df), p = 0.070	43573.978	43777.287	43669.257	0.025 (0.000,0.047)	0.971	0.999	0.997	0.005
**2. Weak measurement invariance (acq2-acq5)** †	30	17.615 (10df), p = 0.062	43570.821	43750.211	43654.890	0.023 (0.000,0.040)	0.997	0.999 (0.000)	0.998	0.011
**2b. Weak measurement invariance (acq1)** †	33	12.697 (7df), p = 0.080	43572.451	43769.780	43664.927	0.024 (0.000,0.044)	0.986	0.999 (0.000)	0.998	0.006
**3. Strong measurement invariance**	26	22.067 (14df), p = 0.077	43567.117	43722.588	43639.977	0.020 (0.000,0.035)	1.000	0.999 (0.000)	0.998	0.010
**4. Factor means invariance**	25	31.527 (15df), p = 0.008	43575.649	43725.141	43645.707	0.027 (0.014,0.041)	0.998	0.998 (-0.001)	0.997	0.031
**Sex (Male, female)**										
**Model**	**No free parameters**	**Chi-square (df), p-value**	**AIC**	**BIC**	**SSA-BIC**	**RMSEA (90%CI)**	**p-close fit**	**CFI (ΔCFI** ‡ **)**	**TLI**	**SRMR**
**1.Configural invariance**	34	13.207 (6df), p = 0.040	43961.088	44164.744	44056.714	0.029 (0.006,0.050)	0.954	0.999	0.997	0.005
**2. Weak measurement invariance (acq2-acq5)** †	30	19.988 (10df), p = 0.030	43958.745	44138.442	44043.120	0.026 (0.008,0.043)	0.993	0.999 (0.000)	0.997	0.012
**2b. Weak measurement invariance (acq1)** †	33	16.723 (7df), p = 0.019	43962.765	44160.432	44055.579	0.031 (0.012,0.050)	0.951	0.999 (0.000)	0.996	0.010
**3. Strong measurement invariance**	26	56.191 (14df), p<0.001	43990.759	44146.496	44063.885	0.045 (0.033,0.058)	0.718	0.994 (-0.005)	0.992	0.017
**3b. Partial scalar invariance** ††	27	27.563 (13df), p = 0.010	43960.647	44122.374	44036.585	0.028 (0.013,0.042)	0.996	0.998 (-0.001)	0.997	0.014
**4. Factor means invariance** †††	25	116.118 (15df), p<0.001	44055.359	44205.106	44125.672	0.068 (0.056,0.079)	0.005	0.986 (-0.013)	0.981	0.068

ACQ: Asthma Control Questionnaire; AIC: Akaike information criterion; BIC: Bayesian Information Criterion; CFI: Comparative Fit Index; df: degrees of freedom; RMSEA: Root Mean Square Error of Approximation; SRMR: Standardised Root Mean Square Residual; SSA-BIC: Sample-Size Adjusted Bayesian Information Criterion; TLI: Tucker-Lewis Index

‡ relative to configural model

† items are assessed separately, with factor loading for acq1 fixed at 1 in model 2 and factor loading for acq2 fixed at 1 in model 2b

†† strong measurement invariance for acq2-acq5 only (not acq1)

††† assuming strong measurement invariance (Model 3), on the grounds that approximate measurement invariance by Bayesian Structural Equation Modelling is accepted

(posterior predictive p-value = 0.196; 95% confidence interval for difference between observed and replicated chi-square values (-10.982,32.227))

Residual correlations (Caucasians, non-Caucasians): acq4 with acq3, acq2 with acq1

Residual correlations (male, female): acq4 with acq3, acq2 with acq1

**Table 4:** Model fit statistics for tests of factorial invariance of the ACQ5 (age).

Age (18–34, 35–54, ≥55 years)										
Model	No. free parameters	Chi-square (df), p-value	AIC	BIC	SSA-BIC	RMSEA (90%CI)	p-close fit	CFI (ΔCFI‡)	TLI	SRMR
**1.Configural invariance**	51	19.874 (9df), p = 0.019	43934.628	44240.113	44078.066	0.035 (0.014,0.056)	0.870	0.999	0.995	0.006
**2. Weak measurement invariance (acq2-acq5)** †	43	38.589 (17df), p = 0.002	43935.873	44193.439	44056.812	0.036 (0.021,0.051)	0.936	0.997 (-0.002)	0.995	0.021
**2b. Weak measurement invariance (acq1)** †	49	31.546 (11df), p<0.001	43943.664	44237.169	44071.478	0.044 (0.026,0.062)	0.696	0.997 (-0.002)	0.992	0.018
**3. Strong measurement invariance**	35	117.188 (25df), p<0.001	44003.944	44213.591	44102.383	0.061 (0.050,0.073)	0.046	0.987 (-0.012)	0.985	0.030
**3b. Partial measurement invariance** ††	37	60.288 (23df), p<0.001	43946.226	44167.852	44050.289	0.041 (0.028,0.053)	0.884	0.995 (-0.004)	0.993	0.026
**4. Factor means invariance** †††	33	179.832 (27df), p<0.001	44067.671	44265.338	44160.485	0.076 (0.066,0.087)	<0.001	0.979 (-0.020)	0.977	0.075

ACQ: Asthma Control Questionnaire; AIC: Akaike information criterion; BIC: Bayesian Information Criterion; CFI: Comparative Fit Index; df: degrees of freedom; RMSEA: Root Mean Square Error of Approximation; SRMR: Standardised Root Mean Square Residual; SSA-BIC: Sample-Size Adjusted Bayesian Information Criterion; TLI: Tucker-Lewis Index

‡ relative to configural model

† items are assessed separately, with factor loading for acq1 fixed at 1 in model 2 and factor loading for acq2 fixed at 1 in model 2b

†† strong measurement invariance for acq2-acq4 only (not acq1)

††† assuming strong measurement invariance (Model 3), on the grounds that approximate measurement invariance by Bayesian Structural Equation Modelling is accepted (posterior predictive p-value = 0.099; 95% confidence interval for difference between observed and replicated chi-square values (-10.284,50.099))

Residual correlations (18–34 years, 35–54 years, ≥55 years): acq4 with acq3, acq2 with acq1

#### 3.3.3 Strong measurement invariance

Average responses to individual items did not vary between Caucasian and non-Caucasian patients with the same absolute levels of asthma symptom control. Reliability was estimated at 93.7% for Caucasians and 90.1% for non-Caucasians. On average responses to items acq2-acq5 did not vary between men and women with the same degree of asthma symptom control. There was some evidence of a possible difference in responses to acq1; women reported being woken more during the night because of their asthma than men with the same level of symptom control (standardised difference in intercepts 0.14 (p<0.001); Table 3, Model 3b). When the invariance model was refit using BSEM, a test of approximate measurement invariance indicated adequate fit to the data (positive predictive p-value = 0.20, 95% Confidence Interval (CI) for the difference between observed and replicated chi-square values [-10.98,32.23]). Reliability was estimated at 89.6% for males and 88.6% for females.

A strong measurement invariance model for age was a poor fit as evidenced by the higher SSA-BIC (44102.38) compared to the configural model (44078.07), a change in the CFI >0.01 compared to the configural model (0.012), an RMSEA above 0.05 (0.06) and p-close fit = 0.05. Inspection of the modification indices showed that non-invariance was due to item1 (Table 4, Model 3b). For patients with the same levels of asthma symptom control, average responses to question acq1 declined with age (intercepts (acq1): 18–34 years (0.16), 35–54 years (0.00), ≥55 years (-0.11). However approximate measurement invariance, assessed using BSEM, was accepted (positive predictive p-value = 0.10, 95% Confidence Interval (CI) for the difference between observed and replicated chi-square values [-10.28,50.10]). Model-based estimates of reliability were 90.9% (18–34 years), 90.5% (35–54 years) and 89.7% (≥55 years).

#### 3.3.4 Comparison of latent factor means

**Fig 3:**
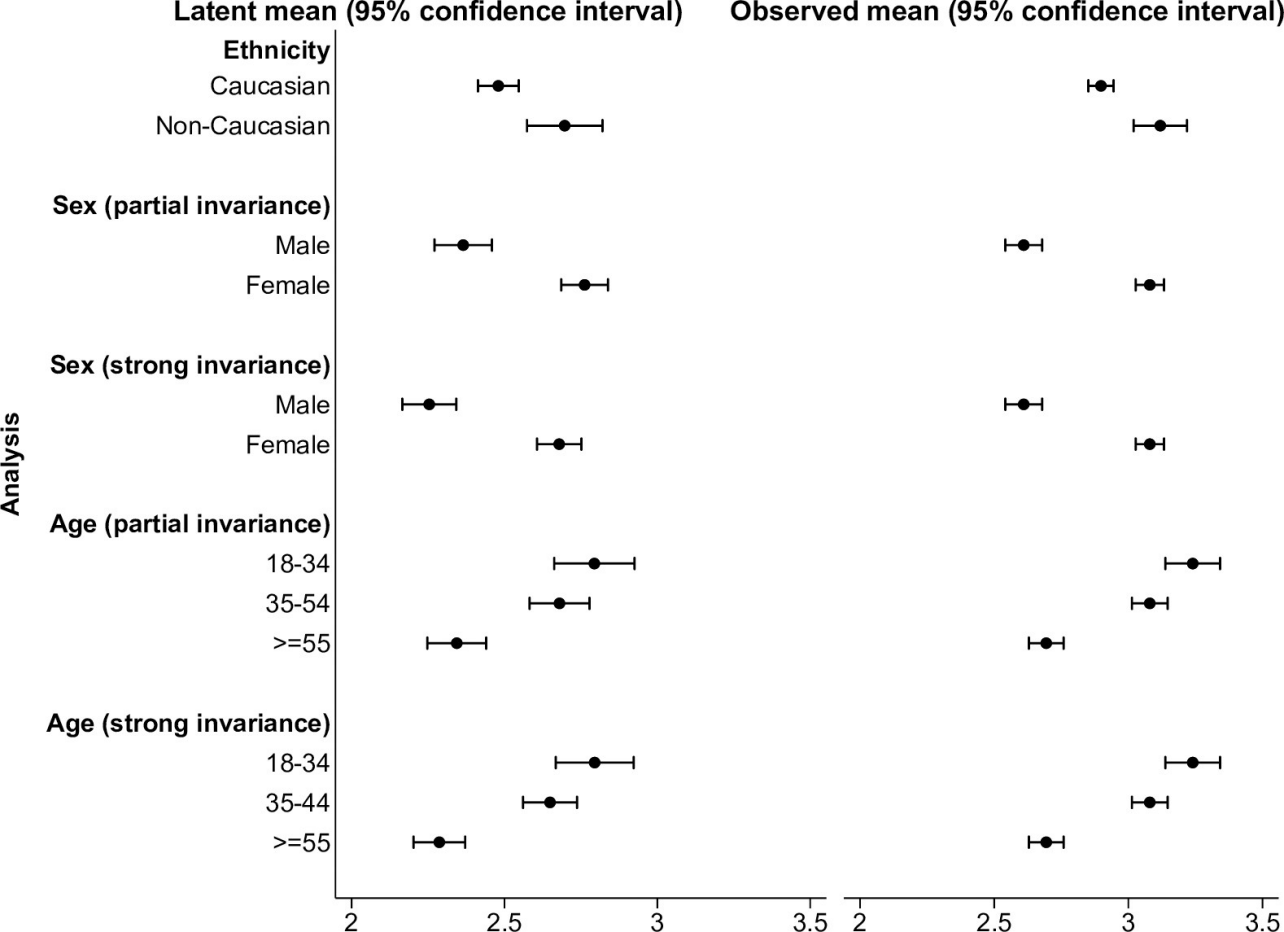
Comparison of latent factor means and estimated means for the ACQ5 among severe asthmatic patients. Higher values for latent factor means indicate higher levels of lack of asthma symptom control.

Fig 3 plots the estimated factor means and the mean ACQ5 scores calculated across the five items. Assuming strong measurement invariance, factor mean levels of lack of asthma symptom control were estimated to be 0.22 (SE 0.07) units higher for non-Caucasians than Caucasians (p = 0.001), 0.43 (SE 0.05) units higher for women than men (p<0.01), and declined with age (18–34 years 2.80 (SE 0.07) units, 35–54 years 2.65 (SE 0.05) units, ≥55 years 2.29 (SE 0.04) units, p<0.01).

The same trends in factor means across sex and age were observed when partial measurement invariance was preferred (sex: 0.40 (SE 0.05) units higher for women than men (p<0.01); age (18–34 years 2.79 (SE 0.07) units, 35–54 years 2.68 (SE 0.05) units, ≥55 years 2.34 (SE 0.05) units, p<0.01)).

Differences in factor mean levels of asthma symptom control mirrored those observed using ACQ5 summary scores.

### 3.4 Sensitivity analyses

#### 3.4.1 Ethnicity and age

When analyses were repeated using all six ethnic groups strong measurement invariance was observed. Mean levels of asthma symptom control differed between Caucasians and the other ethnicities (p<0.001) (Supplementary Materials S5 Table and S1 Fig in S1 File). Results from analyses when age was dichotomised (18–45 years, ≥46 years) or treated as a continuous variable mirrored the main analysis (S6 and S7 Tables and S1 Fig in S1 File).

#### 3.4.2 Clustered data

When the main analyses were rerun to account for clustering of patients within sites, partial measurement invariance was observed for sex and age. There was the possibility of some inaccurate standard errors due to a non-positive definite first-order derivative product matrix (S8-S10 Tables in S1 File).

#### 3.4.3 Levels of type-2 inflammation

Strong measurement invariance held when comparing patients with high levels of type-2 inflammation (blood eosinophil count ≥0.15 10^9^/L and FeNO > = 0.25 ppb) and other patients (S11 Table in S1 File).

#### 3.4.4 ACQ6

The sixth item of the ACQ (acq6) was highly correlated with the latent factor in all analyses (standardised factor loading > = 0.75, p<0.01). Invariance findings from the ACQ5 were replicated, with the exception that approximate measurement invariance was not accepted for age due to non-invariance associated with items acq1 and acq6 (posterior predictive p-value <0.01; 95% confidence interval for difference between observed and replicated chi-square values (22.26,88.67)). Average responses to questions acq1 and acq6 among patients with the same level of asthma symptom control declined with age (intercepts (acq1): 18–34 years (0.16), 35–54 years (0.00), ≥55 years (-0.11); intercepts (acq6): 18–34 years (0.34), 35–54 years (0.19), ≥55 years (0.04)) (S12-S15 Tables and S2 Fig in S1 File).

## 4. Discussion

### 4.1 Principal findings

In this study, we used CFA to examine whether a hypothesised relationship between the questionnaire items of the ACQ5 and a latent factor for asthma symptom control was observed among patients with severe asthma. A one-factor model, which incorporated appropriate residual correlations, was an excellent fit to the data among all subgroups studied. The data was consistent with the hypotheses that the same construct was being measured among participants of different ethnicities, sexes and ages and was being measured on the same scale. On average, responses to individual questionnaire items were the same for Caucasian and non-Caucasian patients with the same level of asthma symptom control. Females and younger patients reported more sleep disturbance due to asthma than males or older patients respectively with the same degree of asthma control; however approximate measurement invariance was observed and this did not have a substantive impact when comparing the factor means of patients by age. Mean levels of lack of asthma control were significantly higher for non-Caucasians, women and younger patients. Reliability of the scale was high in all subgroups studied.

### 4.2 Context of other studies

A small number of studies have assessed invariance of measurement instruments specific to asthma [15, 16, 42, 43]. Measurement invariance of the ACQ6 has been assessed with regards to sex and age among children [15] and over time among adults [16]. However no such studies of the ACQ have been undertaken among severe asthmatic patients.

In this study strong measurement invariance of the ACQ5 was observed when comparing severe asthmatic patients from different ethnicities. Invariance of the ACQ with respect to ethnicity has not been reported elsewhere, although partial measurement invariance was observed when QoL was compared between African-American and Latino patients using the mini-AQLQ [43].

There was some evidence of systematically differing responses to the first questionnaire item of the ACQ5 between sexes with the same degree of asthma symptom control, however average responses were considered approximately equal, consistent with the literature [15]. This is an important finding as some studies report that females have a worse perception of asthma and experience it as more symptomatic than men [44]. Strong measurement invariance of the ACQ5 was not observed when comparing patients of different ages. A study of measurement invariance of the ACQ6 among children concluded that lack of measurement invariance was due to the fifth question [15]. In our study we observed that failure to observe strong measurement invariance was due to the first question. A study of the Jenkins Sleep Scale 4 concluded that questionnaire items ascertaining the extent to which adults reported waking during the night or having trouble staying asleep were not invariant with age [45]. It is possible that our finding may reflect a general issue relating to questions assessing sleep and/or the possibility that patients with severe asthma experience different types of sleeping problems dependent on age. However, the difference in the intercept for acq1 between the oldest and youngest age-group was only 0.27 (Yan et al. considered a difference of 0.19 to be small [15]).

Given that strong or approximate measurement invariance was observed in our analyses, it is not surprising that the unobserved factor means closely mirrored observed average ACQ5 summary scores in terms of relative differences between subgroups. Consequently differences observed between subgroups in mean factor levels of asthma control are consistent with analyses undertaken elsewhere using summary scores only. The subgroups in our analyses with poorer average levels of asthma control were non-Caucasians [46], women [44]and younger adults [47], consistent with the literature. Possible reasons for lower levels of asthma control among patients from ethnic minority groups include poor adherence, lack of engagement with health care systems and higher levels of atopic comorbidities and corticosteroid comorbidities [46]. It has been suggested that factors such as obesity, mood disturbance and hormonal changes may be associated with poorer asthma control among females than males [48, 49]. Older patients are known to have better levels of adherence to medications across the spectrum of asthma severity and this may account in part for the higher levels of asthma control observed in these patients [50]. It may also be associated with differences in asthma phenotype, with the early onset phenotype (associated with higher levels of hospital attendance and exacerbations compared to other phenotypes) more likely to be prevalent among younger adults referred to specialist clinics [8]. Estimated differences in factor means between subgroups of patients were largest for sex (0.49 units) and age (0.51 units). The minimum clinically important difference (MCID) for the ACQ is 0.5 [51]; given the acceptance of approximate measurement invariance this would suggest that these differences in asthma symptom control are clinically relevant.

### 4.3 Strengths and limitations of study

This is the first study which assessed measurement invariance of the ACQ5 among patients with severe asthma and compared subgroups of patients according to common demographic factors, using a large high-quality national database where patients met the criteria for severe asthma using widely accepted guidelines. We followed an accepted methodology for model selection and assessment of goodness-of-fit. Multiple sensitivity analyses were undertaken and these had little material impact on any conclusions drawn. Although the UKSAR population is biased towards patients with type-2 inflammation, measurement invariance was still observed.

There are a number of limitations to this study. Our analyses are susceptible to observational data biases such as confounding due to unmeasured or poorly measured factors, selection bias (e.g. variation between centres in relation to upload of patients to the registry or to whom the ACQ was administered) and information bias (e.g. patient characteristics which are missing or misclassified). To date no “gold standard” measure of asthma symptom control exists and UKSAR does not contain other asthma control questionnaires with which we can compare our results (e.g. ACT). We were also unable to compare our measurement models with patients who have milder asthma using this database. Despite these limitations our findings were consistent with studies examining measurement invariance among other types of asthma patient [15, 16].

### 4.4 Implications for policy and practice

GINA recommends that asthma treatment is titrated against patient symptoms and hence valid ways to measure these symptoms, which perform consistently across all patients, is crucial.

The acceptance of strong/approximate measurement invariance implies that levels of asthma symptom control, estimated using the ACQ5, can be meaningfully compared between severe asthma patients of different ethnicities, sexes or ages using summary scores. It is important that attempts to replicate these studies are undertaken in other severe asthma registries.

## 5. Conclusion

The ACQ5 can be considered to perform equivalently among severe asthmatic patients of different ethnicities, sexes and ages. Consequently comparisons in levels of asthma symptom control made between subgroups of these patients using the ACQ5 are likely to be valid and informative.

## Supporting information

S1 File (PDF)

## Abbreviations

ACQ: Asthma Control Questionnaire
acqi: question/item i of the Asthma Control Questionnaire (i = 1,…,6
ACT: Asthma Control Tes
BIC: Bayesian Information Criterion
BMI: Body Mass Index
BSEM: Bayesian Structural Equation Modelling
CFA: Confirmatory Factor Analysis
CFI: Comparative Fit Index
CI: Confidence Interval
ERS/ATS: European Respiratory Society/American Thoracic Society
FEV_1_: Forced Expiratory Volume in 1 second
FeNO: Fractional Exhaled Nitric Oxide
GINA: Global Initiative for Asthma
GLI: Global Lung Initiative
IQR: Interquartile Range
MAR: Missing At Random
MCID: Minimally Clinically Important Difference
MI: Modification Indices
MIMIC: Multiple Indicator Multiple Causes
MLR: Maximum Likelihood Robust
ORECNI: Office for Research Ethics Committees Northern Ireland
ppb: parts per billion
PROMs: Patient Reported Outcome Measures
QoL: Quality of Life
RCP3Q: Royal College of Physicians Three Questions
RMSEA: Root Mean Square Error of Approximation
SABA: Short-Acting Beta Agonists
SAQ: Severe Asthma Questionnaire
SD: Standard Deviation
SMART: Single Inhaler Maintenance and Reliever Therapy
SRMR: Standardised Root Mean Square Residual
SSA-BIC: Sample Size Adjusted-Bayesian Information Criterion
TLI: Tucker Lewis Index
UKSAR: United Kingdom Severe Asthma Registry
