# Supplementary material for: An examination of factorial invariance of the Asthma Control Questionnaire among adults with severe asthma
Source: PLoS One. 2023 Dec 7;18(12):e0295493. doi: 10.1371/journal.pone.0295493 (PMC10703262; doi:10.1371/journal.pone.0295493)

An examination of factorial invariance of the Asthma Control Questionnaire among adults with severe asthma

Supplementary Materials

Index of Legends

Tables

S1 Table	Comparison of patient characteristics by ethnicity
S2 Table	Comparison of patient characteristics by sex
S3 Table	Comparison of patient characteristics by age-group
S4 Table	Preliminary examination of factorial invariance of the ACQ5 (whole sample)
S5 Table	Sensitivity analysis: tests of factorial invariance of the ACQ5 (ethnicity)
S6 Table	Sensitivity analysis: tests of factorial invariance of the ACQ5 (age)
S7 Table	Sensitivity analysis: test of factorial invariance of the ACQ5 (age) using a Multiple Indicator Multiple Outcomes (MIMIC) model
S8 Table	Sensitivity analysis: tests of factorial invariance of the ACQ5 (ethnicity), accounting for clustering of patients within sites
S9 Table	Sensitivity analysis: tests of factorial invariance of the ACQ5 (sex), accounting for clustering of patients within sites
S10 Table	Sensitivity analysis: tests of factorial invariance of the ACQ5 (age), accounting for clustering of patients within sites
S11 Table	Sensitivity analysis: tests of factorial invariance of the ACQ5 (type-2 inflammation)
S12 Table	Mean (SD) ACQ6 item scores by sex, ethnicity and age
S13 Table	Sensitivity analysis: tests of factorial invariance of the ACQ6 (ethnicity)
S14 Table	Sensitivity analysis: tests of factorial invariance of the ACQ6 (sex)
S15 Table	Sensitivity analysis: tests of factorial invariance of the ACQ6 (age)

Figures

S1 Fig.	Sensitivity analysis: comparison of latent factor means and estimated means for the ACQ5 among severe asthmatic patients
S2 Fig.	Sensitivity analysis: Comparison of latent factor means and estimated means for the ACQ6 among severe asthmatic patients

S1 Table. Comparison of patient characteristics by ethnicity

Variable	Caucasian (n=2,369)	Non-Caucasian (n=240)	p-value‡
**Demographic variables**			
**Sex**			
Female	1,440 (60.8%)	358 (64.9%)	0.077
Male	929 (39.2%)	194 (35.1%)	
**Age at presentation (years)**			
18-34	414 (17.5%)	90 (16.3%)	<0.001
35-54	885 (37.4%)	271 (49.1%)	
≥55	1,070 (45.2%)	191 (34.6%)	
**Age of onset (years)**			
<12	745 (35.2%)	171 (33.0%)	0.402
12-18	219 (10.3%)	48 (9.3%)	
>18	1,154 (54.5%)	299 (57.7%)	
**Smoking status**			
Never	1,485 (63.7%)	412 (75.6%)	<0.001
Ex-smoker	735 (31.5%)	116 (21.3%)	
Current smoker	113 (4.8%)	17 (3.1%)	
**BMI (kg-m2)**	30.9 (7.3)	30.0 (6.4)	0.002
**Clinical measures**			
**Clinic FEV_1 (% Predicted)**	67.7 (21.0)	64.0 (20.5)	<0.001
**Clinic FVC (% Predicted)**	84.4 (18.8)	80.8 (19.8)	<0.001
**Clinic FEV_1/FVC**	64.5 (17.3)	65.2 (16.1)	0.410
**Blood eosinophil count (10^9/L) ***	0.36 (0.20,0.60)	0.40 (0.20,0.60)	0.003
**Highest blood eosinophil count† (10^9/L) ***	0.65 (0.40,1.00)	0.60 (0.40,1.00)	0.386
**FeNO (ppb) ***	39 (20,72)	45 (27,81)	0.001
**Total IgE (IU/mL) ***	135 (47,385)	244 (93,597)	<0.001
**Medication and service use**			
**On maintenance oral corticosteroids**	1,177 (50.0%)	253 (45.9%)	0.081
**Exacerbations requiring rescue steroids In previous year ***	4 (2,7)	4 (2,8)	0.495
**Invasive ventilations (Ever)**	207 (9.4%)	64 (12.1%)	0.056
**Emergency department visit/ hospital admission in previous year**	1,058 (46.7%)	306 (56.98%)	<0.001

Categorical variables summarised as counts (%); scalar measures reported as mean (standard deviation) unless otherwise indicated

* median, inter-quartile range reported due to skewed distribution of scalar measure
‡ chi-squared test (categorical variables): t-test (unequal variances) or Wilcoxon Rank-Sum Test (continuous variables)
† as recorded in medical records.

BMI: Body Mass Index; FEV_1: FeNO: Fractional Exhaled Nitric Oxide; Forced Expiratory Volume in one second; FVC: Forced Vital Capacity; FeNO: Fractional Exhaled Nitric Oxide; IgE: Immunoglobulin E

S2 Table. Comparison of patient characteristics by sex

Variable	Male (n=1,130)	Female (n=1,821)	p-value ‡
**Demographic variables**			
**Age at presentation (years)**			
18-34	129 (11.4%)	382 (21.0%)	<0.001
35-54	431 (38.1%)	736 (40.4%)	
≥55	570 (50.4%)	703 (38.6%)	
**Age of onset (years)**			
<12	325 (32.3%)	602 (36.3%)	<0.001
12-18	56 (5.6%)	214 (12.9%)	
>18	625 (62.1%)	842 (50.8%)	
**Ethnicity**			
Caucasian	929 (82.7%)	1,440 (80.1%)	0.077
Non-Caucasian	194 (17.3%)	358 (19.9%)	
**Smoking status**			
Never	692 (62.1%)	1,224 (68.3%)	<0.001
Ex-smoker	385 (34.5%)	474 (26.5%)	
Current smoker	38 (3.4%)	94 (5.2%)	
**BMI (kg-m2)**	29.7 (5.8)	31.5 (7.8)	<0.001
**Clinical measures**			
**Clinic FEV_1 (% Predicted)**	64.7 (20.9)	68.4 (20.9)	<0.001
**Clinic FVC (% Predicted)**	84.3 (18.9)	83.4 (19.2)	0.244
**Clinic FEV_1/FVC**	61.3 (20.8)	66.8 (13.9)	<0.001
**Blood eosinophil count (10^9/L) ***	0.38 (0.20,0.60)	0.39 (0.20,0.60)	0.688
**Highest blood eosinophil count † (10^9/L) ***	0.67 (0.40,1.10)	0.61 (0.40,1.00)	0.168
**FeNO (ppb) ***	47 (26,82)	36.9 (19,68)	<0.001
**Total IgE (IU/mL) ***	196 (75,540)	127 (42,347)	<0.001
**Medication and service use**			
**On maintenance oral corticosteroids**	588 (52.4%)	856 (47.3%)	0.007
**Exacerbations requiring rescue steroids In previous year ***	4 (2,6)	5 (3,8)	<0.001
**Invasive ventilations (Ever)**	92 (8.8%)	183 (10.6%)	0.132
**Emergency department visit/ hospital admission in previous year**	444 (41.5%)	933 (53.0%)	<0.001

Categorical variables summarised as counts (%); scalar measures reported as mean (standard deviation) unless otherwise indicated

* median, inter-quartile range reported due to skewed distribution of scalar measure
‡ chi-squared test (categorical variables): t-test (unequal variances) or Wilcoxon Rank-Sum Test (continuous variables)
† as recorded in medical records.

BMI: Body Mass Index; FEV_1: FeNO: Fractional Exhaled Nitric Oxide; Forced Expiratory Volume in one second; FVC: Forced Vital Capacity; FeNO: Fractional Exhaled Nitric Oxide; IgE: Immunoglobulin E

S3 Table. Comparison of patient characteristics by age-group

Variable	18-34 (n=511)	35-54 (n=1,167)	≥55 years (n=1,240)	p-value‡
**Demographic characteristics**				
**Sex**				
Female	382 (74.8%)	736 (63.1%)	703 (55.2%)	<0.001
Male	129 (25.2%)	431 (36.9%)	570 (44.8%)	
**Age of Onset (years)**				
<12	299 (65.0%)	374 (35.2%)	254 (22.2%)	<0.001
12-18	78 (17.0%)	120 (11.3%)	72 (6.3%)	
>18	83 (18.0%)	568 (53.5%)	816 (71.5%)	
**Ethnicity**				
Caucasian	414 (82.1%)	885 (76.6%)	1,070 (84.9%)	<0.001
Non-Caucasian	26 (5.2%)	271 (23.4%)	191 (15.1%)	
**Smoking Status**				
Never	363 (72.0%)	722 (62.9%)	363 (72.0%)	<0.001
Ex-smoker	91 (18.1%)	376 (32.8%)	91 (18.1%)	
Current smoker	50 (9.9%)	50 (4.4%)	50 (9.9%)	
**BMI (kg-m2)**	30.3 (8.0)	31.6 (7.6)	30.3 (6.3)	0.926
**Clinical measures**				
**Clinic FEV_1 (% Predicted)**	72.7 (19.9)	66.1 (20.7)	65.5 (21.3)	<0.001
**Clinic FVC (% Predicted)**	88.5 (17.4)	82.2 (18.7)	83.2 (19.7)	<0.001
**Clinic FEV_1/FVC**	69.7 (13.1)	65.1 (14.3)	62.4 (20.1)	<0.001
**Blood eosinophil count ($N/10^9L$) ***	0.40 (0.19,0.60)	0.39 (0.20,0.60)	0.36 (0.20,0.60)	0.133
**Highest blood eosinophil count † ($N/10^9L$) ***	0.70 (0.40,1.10)	0.60 (0.40,1.00)	0.64 (0.40,1.00)	0.666
**FeNO (ppb) ***	42 (19,78)	40 (20,76)	40 (22,69)	0.526
**Total IgE (IU/mL) ***	209 (76,597)	140 (52,374)	140 (48,399)	<0.001
**Medication and service use**				
**On maintenance oral corticosteroids**	199 (39.0%)	565 (48.7%)	680 (53.9%)	<0.001
**Exacerbations requiring rescue steroids In previous year ***	5 (3,8)	4 (3,7)	4 (2,6)	<0.001
**Invasive ventilations (Ever)**	69 (14.5%)	122 (11.1%)	84 (7.1%)	<0.001
**Emergency department visit/ hospital admission in previous year**	326 (65.7%)	575 (51.5%)	476 (39.0%)	<0.001

Categorical variables summarised as counts (%); scalar measures reported as mean (standard deviation) unless otherwise indicated

* median, inter-quartile range reported due to skewed distribution of scalar measure
‡ chi-squared test (categorical variables): ANOVA or non-parametric test for trend - ordered groups (continuous variables)
† as recorded in medical records.

BMI: Body Mass Index; FEV_1: FeNO: Fractional Exhaled Nitric Oxide; Forced Expiratory Volume in one second; FVC: Forced Vital Capacity; FeNO: Fractional Exhaled Nitric Oxide; IgE: Immunoglobulin E

S4 Table. Preliminary examination of factorial invariance of the ACQ5 (whole sample)

Ethnicity (Caucasian, SE Asian, NE Asian, African, Mixed, Other)										
**Model**	**No free parameters**	**Chi-square (df), p-value**	**AIC**	**BIC**	**SSA-BIC**	**RMSEA (90%CI)**	**p-close fit**	**CFI (ΔCFI‡)**	**TLI**	**SRMR**
**1.Configural invariance**	15	335.986 (5df), p<0.001	44372.355	44462.204	44414.543	0.130 (0.112,0.144)	0.001	0.970	0.941	0.018
**2. Add residual correlation between acq3 and acq4 to Model 1**	16	59.447 (4df), p<0.001	44119.949	44215.788	44164.950	0.069 (0.054,0.084)	0.020	0.992 (0.022)	0.981	0.011
**3. Add residual correlation between acq1 and acq2 to Model 2**	17	12.020 (3df), p=0.007	44056.519	44158.647	44104.632	0.032 (0.015,0.052)	0.931	0.999 (0.029)	0.996	0.005

ACQ: Asthma Control Questionnaire; AIC: Akaike information criterion; BIC: Bayesian Information Criterion; CFI: Comparative Fit Index; df: degrees of freedom; RMSEA: Root Mean Square Error of Approximation ; SRMR: Standardised Root Mean Square Residual; SSA-BIC: Sample-Size Adjusted Bayesian Information Criterion; TLI: Tucker-Lewis Index

‡ relative to configural model

S5 Table. Sensitivity analysis: tests of factorial invariance of the ACQ5 (ethnicity)

Ethnicity (Caucasian, SE Asian, NE Asian, African, Mixed, Other)										
**Model**	**No free parameters**	**Chi-square (df), p-value**	**AIC**	**BIC**	**SSA-BIC**	**RMSEA (90%CI)**	**p-close fit**	**CFI (ΔCFI‡)**	**TLI**	**SRMR**
**1.Configural invariance**	96	22.352 (24df), p=0.558	43595.923	44169.972	43864.945	0.000 (0.000,0.034)	0.998	1.000	1.001	0.007
**2. Weak measurement invariance (acq2-qcq5) †**	76	49.882 (44df), p=0.251	43582.766	44037.222	43795.742	0.017 (0.000,0.036)	0.999	0.999 (-0.001)	0.999	0.026
**2b. Weak measurement invariance (acq1) †**	91	26.927 (29df), p=0.576	43589.776	44133.927	43844.786	0.000 (0.000,0.031)	0.999	1.000 (0.000)	1.001	0.012
**3. Strong measurement invariance**	56	61.742 (64df), p=0.557	43554.155	43889.017	43711.084	0.000 (0.000,0.025)	1.000	1.000 (0.000)	1.000	0.025
**4. Factor means invariance**	51	75.186 (69df), p=0.285	43558.518	43863.482	43701.436	0.014 (0.000,0.031)	1.000	0.999 (-0.001)	0.999	0.045
**4b. Different factor mean (Caucasians)**	52	64.335 (68df), p=0.604	43548.653	43859.596	43694.373	0.000 (0.000,0.024)	1.000	1.000 (0.000)	1.000	0.029

ACQ: Asthma Control Questionnaire; AIC: Akaike information criterion; BIC: Bayesian Information Criterion; CFI: Comparative Fit Index; df: degrees of freedom; RMSEA: Root Mean Square Error of Approximation ; SRMR: Standardised Root Mean Square Residual; SSA-BIC: Sample-Size Adjusted Bayesian Information Criterion; TLI: Tucker-Lewis Index

‡ relative to configural model
† items are assessed separately, with factor loading for acq1 fixed at 1 in model 2 and factor loading for acq2 fixed at 1 in model 2b

Residual correlations (Caucasian): acq4 with acq3, acq2 with acq1
Residual correlations (SE Asian, other): acq4 with acq3
Residual correlations (African): acq2 with acq1
Residual correlations (Mixed): acq3 with acq2

S6 Table. Sensitivity analysis: tests of factorial invariance of the ACQ5 (age)

Age (18-45 years, ≥46 years)										
**Model**	**No free parameters**	**Chi-square (df), p-value**	**AIC**	**BIC**	**SSA-BIC**	**RMSEA (90%CI)**	**p-close fit**	**CFI (ΔCFI‡)**	**TLI**	**SRMR**
**1.Configural invariance**	34	20.050 (6df), p=0.003	43960.793	44164.450	44056.419	0.040 (0.021,0.060)	0.781	0.999	0.996	0.005
**2. Weak measurement invariance (acq2-qcq5) †**	30	30.210 (10df), p=0.001	43962.953	44142.650	44047.329	0.037 (0.022,0.053)	0.913	0.998 (-0.001)	0.996	0.017
**2b. Weak measurement invariance (acq1) †**	33	27.032 (7df), p<0.001	43965.775	44163.442	44055.589	0.044 (0.027,0.062)	0.681	0.998 (-0.001)	0.995	0.013
**3. Strong measurement invariance**	26	99.760 (14df), p<0.001	44024.503	44180.240	44097.629	0.064 (0.053,0.077)	0.021	0.992 (-0.007)	0.989	0.023
**3b. Partial measurement invariance ††**	27	43.132 (13df), p<0.001	43969.875	44131.602	44045.813	0.040 (0.027,0.053)	0.891	0.997 (-0.002)	0.996	0.018
**4. Factor means invariance †††**	25	144.613 (15df), p<0.001	44067.356	44217.104	44137.669	0.077 (0.065,0.088)	<0.001	0.988 (-0.011)	0.984	0.065

ACQ: Asthma Control Questionnaire; AIC: Akaike information criterion; BIC: Bayesian Information Criterion; CFI: Comparative Fit Index; df: degrees of freedom; RMSEA: Root Mean Square Error of Approximation ; SRMR: Standardised Root Mean Square Residual; SSA-BIC: Sample-Size Adjusted Bayesian Information Criterion; TLI: Tucker-Lewis Index

‡ relative to configural model
† items are assessed separately, with factor loading for acq1 fixed at 1 in model 2 and factor loading for acq2 fixed at 1 in model 2b

†† strong measurement invariance for acq2-acq5 only (not acq1)
††† assuming strong measurement invariance (Model 3), on the grounds that approximate measurement invariance by Bayesian Structural Equation Modelling is accepted (posterior predictive p-value p=0.118, 95CI for the difference between observed and replicated chi-square values (-10.167,38.138))

Residual correlations (18-45 years; >=46 years): acq4 with acq3, acq2 with acq1

S7 Table. Sensitivity analysis: test of factorial invariance of the ACQ5 (age) using a Multiple Indicator Multiple Outcomes (MIMIC) model

Age (continuous measure)										
**Model**	**No free parameters**	**Chi-square (df), p-value**	**AIC**	**BIC**	**SSA-BIC**	**RMSEA (90%CI)**	**p-close fit**	**CFI (ΔCFI‡)**	**TLI**	**SRMR**
**1.Baseline model**	18	106.136 (7df), p<0.001	43980.344	44088.163	44030.970	0.069 (0.058,0.081)	0.003	0.988	0.974	0.023
**2. Add direct effect of age on acq1**	19	33.387 (6df), p<0.001	43897.734	44011.542	43951.172	0.039 (0.027,0.053)	0.900	0.997 (+0.009)	0.992	0.010

ACQ: Asthma Control Questionnaire; AIC: Akaike information criterion; BIC: Bayesian Information Criterion; CFI: Comparative Fit Index; df: degrees of freedom; RMSEA: Root Mean Square Error of Approximation ; SRMR: Standardised Root Mean Square Residual; SSA-BIC: Sample-Size Adjusted Bayesian Information Criterion; TLI: Tucker-Lewis Index

‡ relative to configural model

Residual correlations: acq4 with acq1, acq2 with acq1

S8 Table. Sensitivity analysis: tests of factorial invariance of the ACQ5 (ethnicity), accounting for clustering of patients within sites

Ethnicity (Caucasian, non-Caucasian)										
**Model**	**No free parameters**	**Chi-square (df), p-value**	**AIC**	**BIC**	**SSA-BIC**	**RMSEA (90%CI)**	**p-close fit**	**CFI (ΔCFI‡)**	**TLI**	**SRMR**
**1.Configural invariance**	35	7.206 (5df), p=0.206	43573.180	43782.469	43671.261	0.017 (0.000,0.043)	0.986	0.999	0.998	0.004
**2. Weak measurement invariance (acq2-qcq5) †**	31	12.586 (9df), p=0.182	43569.430	43754.800	43656.301	0.017 (0.000,0.036)	0.999	0.999 (0.000)	0.998	0.011
**2b. Weak measurement invariance (acq1) †**	34	8.300 (6df), p=0.217	43571.264	43774.573	43666.543	0.016 (0.000,0.040)	0.994	0.999 (0.000)	0.998	0.004
**3. Strong measurement invariance**	27	14.622 (13df), p=0.332	43565.578	43727.129	43641.340	0.009 (0.000,0.028)	1.000	1.000 (+0.001)	0.999	0.010
**4. Factor means invariance**	26	21.898 (14df), p=0.081	43574.128	43729.599	43646.988	0.020 (0.000,0.035)	1.000	0.998 (-0.001)	0.997	0.031

ACQ: Asthma Control Questionnaire; AIC: Akaike information criterion; BIC: Bayesian Information Criterion; CFI: Comparative Fit Index; df: degrees of freedom; RMSEA: Root Mean Square Error of Approximation ; SRMR: Standardised Root Mean Square Residual; SSA-BIC: Sample-Size Adjusted Bayesian Information Criterion; TLI: Tucker-Lewis Index

‡ relative to configural model
† items are assessed separately, with factor loading for acq1 fixed at 1 in model 2 and factor loading for acq2 fixed at 1 in model 2b

Residual correlations (Caucasians): acq4 with acq3, acq2 with acq1
Residual correlations (non-Caucasians): acq4 with acq3, acq2 with acq1, acq4 with acq1

S9 Table. Sensitivity analysis: tests of factorial invariance of the ACQ5 (sex), accounting for clustering of patients within sites

Sex (male, female)										
**Model**	**No free parameters**	**Chi-square (df), p-value**	**AIC**	**BIC**	**SSA-BIC**	**RMSEA (90%CI)**	**p-close fit**	**CFI (ΔCFI‡)**	**TLI**	**SRMR**
**1.Configural invariance**	34	10.619 (6df), p<0.001	43961.088	44164.744	44156.714	0.023 (0.000,0.045)	0.982	0.999	0.995	0.005
**2. Weak measurement invariance (acq2-qcq5) †**	30	17.625 (10df), p=0.062	43958.745	44138.442	44043.120	0.023 (0.000,0.040)	0.997	0.998 (-0.001)	0.996	0.012
**2b. Weak measurement invariance (acq1) †**	33	13.567 (7df), p=0.059	43962.765	44160.432	44055.579	0.025 (0.000,0.045)	0.982	0.998 (-0.001)	0.995	0.010
**3. Strong measurement invariance**	26	50.356 (14df), p<0.001	43990.759	44146.496	44063.885	0.042 (0.030,0.055)	0.842	0.989 (-0.010)	0.985	0.017
**3b. Partial measurement invariance ††**	27	25.485 (13df), p=0.020	43960.647	44122.374	44036.585	0.026 (0.010,0.040)	0.998	0.996 (-0.003)	0.994	0.014
**4. Factor means invariance ††**	26	76.123 (14df), p<0.001	44018.116	44173.854	44091.242	0.055 (0.043,0.067)	0.236	0.982 (-0.017)	0.974	0.061

ACQ: Asthma Control Questionnaire; AIC: Akaike information criterion; BIC: Bayesian Information Criterion; CFI: Comparative Fit Index; df: degrees of freedom; RMSEA: Root Mean Square Error of Approximation ; SRMR: Standardised Root Mean Square Residual; SSA-BIC: Sample-Size Adjusted Bayesian Information Criterion; TLI: Tucker-Lewis Index

‡ relative to configural model
† items are assessed separately, with factor loading for acq1 fixed at 1 in model 2 and factor loading for acq2 fixed at 1 in model 2b
†† strong measurement invariance for acq2-acq5 only (not acq1); approximate measurement invariance with clustered data cannot be assessed using Bayesian Structural Equation Modelling

Residual correlations (male, female): acq4 with acq3, acq2 with acq1

S10 Table. Sensitivity analysis: test of factorial invariance of the ACQ5 (age), accounting for clustering of patients within sites

Age (18-34, 35-54, ≥55 years)										
**Model**	**No. free parameters**	**Chi-square (df), p-value**	**AIC**	**BIC**	**SSA-BIC**	**RMSEA (90%CI)**	**p-close fit**	**CFI (ΔCFI‡)**	**TLI**	**SRMR**
**1.Configural invariance**	51	22.403 (9df), p=0.008	43934.628	44240.113	44078.066	0.039 (0.019,0.059)	0.798	0.997	0.990	0.006
**2. Weak measurement invariance (acq2-qcq5) †**	43	39.984 (17df), p=0.001	43935.873	44193.439	44056.812	0.037 (0.022,0.052)	0.919	0.995 (-0.002)	0.991	0.021
**2b. Weak measurement invariance (acq1) †**	49	33.887 (11df), p<0.001	43943.664	44237.169	44081.478	0.046 (0.029,0.064)	0.615	0.995 (-0.002)	0.986	0.018
**3. Strong measurement invariance**	35	111.443 (25df), p<0.001	44003.944	44213.591	44102.383	0.059 (0.048,0.071)	0.080	0.980 (-0.017)	0.976	0.030
**3b. Partial measurement invariance ††**	37	63.113 (23df), p<0.001	43946.226	44167.852	44050.289	0.042 (0.030,0.055)	0.841	0.991 (-0.006)	0.988	0.026
**4. Factor means invariance ††**	35	122.348 (25df), p<0.001	43999.749	44209.395	44098.187	0.063 (0.052,0.074)	0.026	0.978 (-0.019)	0.973	0.064
**4b. Different latent mean for ages ≥55 years††**	36	66.992 (24df), p<0.001	43946.807	44162.443	44048.057	0.043 (0.031,0.055)	0.829	0.990 (-0.007)	0.988	0.028

ACQ: Asthma Control Questionnaire; AIC: Akaike information criterion; BIC: Bayesian Information Criterion; CFI: Comparative Fit Index; df: degrees of freedom; RMSEA: Root Mean Square Error of Approximation ; SRMR: Standardised Root Mean Square Residual; SSA-BIC: Sample-Size Adjusted Bayesian Information Criterion; TLI: Tucker-Lewis Index

‡ relative to configural model
† items are assessed separately, with factor loading for acq1 fixed at 1 in model 2 and factor loading for acq2 fixed at 1 in model 2b
†† strong measurement invariance for acq2-acq4 (not acq1 or acq5); approximate measurement invariance with clustered data cannot be assessed using Bayesian Structural Equation Modelling

Residual correlations (18-34 years, 35-54 years, >=55 years): acq4 with acq3, acq2 with acq1

S11 Table. Sensitivity analysis: tests of factorial invariance of the ACQ5 (type-2 inflammation)

Type-2 inflammation (high: blood eosinophil count ≥0.15 10^9/L & FeNO >=0.25 ppb; all other patients)										
**Model**	**No. free parameters**	**Chi-square (df), p-value**	**AIC**	**BIC**	**SSA-BIC**	**RMSEA (90%CI)**	**p-close fit**	**CFI (ΔCFI‡)**	**TLI**	**SRMR**
**1.Configural invariance**	34	17.584 (6df), p=0.007	33693.839	33888.515	33780.492	0.041 (0.020,0.064)	0.704	0.998	0.993	0.007
**2. Weak measurement invariance (acq2-acq5) †**	30	25.545 (10df), p=0.004	33691.954	33863.727	33768.415	0.037 (0.019,0.055)	0.872	0.997 (-0.001)	0.994	0.015
**2b. Weak measurement invariance (acq1) †**	33	19.287 (7df), p=0.007	33692.712	33881.663	33776.816	0.039 (0.019,0.061)	0.773	0.998 (0.000)	0.994	0.008
**3. Strong measurement invariance**	26	38.103 (14df), p<0.001	33696.791	33845.661	33763.055	0.039 (0.024,0.054)	0.879	0.996 (-0.002)	0.994	0.021
**4. Factor means invariance**	25	42.142 (15df), p<0.001	33699.009	33842.153	33762.724	0.040 (0.026,0.054)	0.866	0.995 (-0.003)	0.994	0.029

ACQ: Asthma Control Questionnaire; AIC: Akaike information criterion; BIC: Bayesian Information Criterion; CFI: Comparative Fit Index; df: degrees of freedom; RMSEA: Root Mean Square Error of Approximation ; SRMR: Standardised Root Mean Square Residual; SSA-BIC: Sample-Size Adjusted Bayesian Information Criterion; TLI: Tucker-Lewis Index

‡ relative to configural model
† items are assessed separately, with factor loading for acq1 fixed at 1 in model 2 and factor loading for acq2 fixed at 1 in model 2b

Residual correlations (high levels of type-2 inflammation, all other patients): acq4 with acq3, acq2 with acq1

S12 Table. Mean (SD) ACQ6 item scores by sex, ethnicity and age

		Ethnicity			Sex			Age			
**ACQ6 item**	**All patients**	**Caucasian (n=2,369)**	**Non-Caucasian (n=552)**	**p-value**	**Female (n=1,821)**	**Male (n=1,130)**	**p-value**	**18-34 (n=511)**	**35-54 (n=1,167)**	**≥55 years (n=1,273)**	**p-value**
**acq1 (wakening during the night due to asthma)**	2.5 (1.7)	2.5 (1.7)	2.7 (1.7)	0.002	2.8 (1.7)	2.1 (1.7)	<0.001	3.1 (1.7)	2.7 (1.7)	2.1 (1.6)	<0.001
**acq2 (severity of morning symptoms)**	2.9 (1.5)	2.8 (1.4)	3.1 (1.6)	0.002	3.0 (1.4)	2.6 (1.5)	<0.001	3.2 (1.4)	3.0 (1.5)	2.6 (1.4)	<0.001
**acq3 (limitation in activities)**	2.9 (1.5)	2.9 (1.5)	3.1 (1.6)	0.003	3.1 (1.5)	2.7 (1.6)	<0.001	3.1 (1.5)	3.0 (1.5)	2.7 (1.5)	<0.001
**acq4 (shortness of breath)**	3.4 (1.5)	3.3 (1.5)	3.5 (1.5)	0.024	3.5 (1.5)	3.1 (1.6)	<0.001	3.6 (1.5)	3.5 (1.5)	3.2 (1.6)	<0.001
**acq5 (wheezing)**	3.0 (1.6)	2.9 (1.7)	3.2 (1.6)	0.001	3.1 (1.6)	2.8 (1.7)	<0.001	3.2 (1.6)	3.2 (1.6)	2.8 (1.6)	<0.001
**acq6 (use of short-acting bronchodilator)**	2.7 (1.7)	2.7 (1.7)	2.9 (1.7)	0.007	2.9 (1.7)	2.5 (1.7)	<0.001	3.3 (1.8)	2.9 (1.7)	2.3 (1.6)	<0.001

ACQ: Asthma Control Questionnaire; SD standard deviation

S13 Table. Model fit statistics for tests of factorial invariance of the ACQ6 (ethnicity)

Ethnicity (Caucasian, non-Caucasian)										
**Model**	**No free parameters**	**Chi-square (df), p-value**	**AIC**	**BIC**	**SSA-BIC**	**RMSEA (90%CI)**	**p-close fit**	**CFI (ΔCFI‡)**	**TLI**	**SRMR**
**1.Configural invariance**	42	21.98 (12df), p=0.04	52627.88	52879.02	52745.57	0.02 (0.01,0.04)	1.00	1.00	1.00	0.01
**2. Weak measurement invariance (acq2-acq6) †**	37	36.49 (17df), p<0.01	52632.31	52853.55	52735.99	0.03 (0.02,0.04)	1.00	1.00 (0.00)	1.00	0.02
**2b. Weak measurement invariance (acq1) †**	41	22.90 (13df), p=0.04	52626.07	52871.24	52740.97	0.02 (0.00,0.04)	1.00	1.00 (0.00)	1.00	0.01
**3. Strong measurement invariance**	32	40.87 (22df), p=0.01	52625.76	52817.11	52715.44	0.02 (0.01,0.04)	1.00	1.00 (0.00)	1.00	0.02
**4. Factor means invariance**	31	50.68 (23df), p<0.01	52634.93	52820.31	52721.81	0.03 (0.02,0.04)	1.00	1.00 (0.00)	1.00	0.03

ACQ: Asthma Control Questionnaire; AIC: Akaike information criterion; BIC: Bayesian Information Criterion; CFI: Comparative Fit Index; df: degrees of freedom; RMSEA: Root Mean Square Error of Approximation ; SRMR: Standardised Root Mean Square Residual; SSA-BIC: Sample-Size Adjusted Bayesian Information Criterion; TLI: Tucker-Lewis Index

‡ relative to configural model
† items are assessed separately, with factor loading for acq1 fixed at 1 in model 2 and factor loading for acq2 fixed at 1 in model 2b

Residual correlations (Caucasians): acq4 with acq3, acq5 with acq4, acq6 with acq2, acq5 with acq3
Residual correlations (non-Caucasians): acq4 with acq3, acq6 with acq1

S14 Table. Model fit statistics for tests of factorial invariance of the ACQ6 (sex)

Sex (male, female)										
**Model**	**No free parameters**	**Chi-square (df), p-value**	**AIC**	**BIC**	**SSA-BIC**	**RMSEA (90%CI)**	**p-close fit**	**CFI (ΔCFI‡)**	**TLI**	**SRMR**
**1.Configural invariance**	42	21.52 (12df), p=0.04	53109.25	53360.83	53227.38	0.02 (0.00,0.04)	1.00	1.00	1.00	0.01
**2. Weak measurement invariance (acq2-acq6) †**	37	42.52 (17df), p<0.01	53121.52	53343.14	53225.58	0.03 (0.02,0.04)	1.00	1.00 (0.00)	1.00	0.02
**2b. Weak measurement invariance (acq1) †**	41	26.61 (13df), p=0.01	53113.01	53358.60	53227.33	0.03 (0.01,0.01)	1.00	1.00 (0.00)	1.00	0.01
**3. Strong measurement invariance**	32	74.89 (22df), p<0.01	53146.98	53338.65	53236.98	0.04 (0.03,0.05)	0.94	1.00 (0.00)	1.00	0.02
**3b. Partial measurement invariance ††**	33	51.05 (21df), p<0.01	53122.09	53319.75	53214.90	0.03 (0.02,0.04)	1.00	1.00 (0.00)	1.00	0.02
**4. Factor means invariance †††**	31	135.96 (23df), p<0.01	53213.72	53399.40	53300.90	0.06 (0.05,0.07)	0.08	0.99 (-0.01)	0.98	0.07

ACQ: Asthma Control Questionnaire; AIC: Akaike information criterion; BIC: Bayesian Information Criterion; CFI: Comparative Fit Index; df: degrees of freedom; RMSEA: Root Mean Square Error of Approximation ; SRMR: Standardised Root Mean Square Residual; SSA-BIC: Sample-Size Adjusted Bayesian Information Criterion; TLI: Tucker-Lewis Index

‡ relative to configural model
† items are assessed separately, with factor loading for acq1 fixed at 1 in model 2 and factor loading for acq2 fixed at 1 in model 2b
†† strong measurement invariance for acq2-acq6 (not acq1)
††† assuming strong measurement invariance (Model 3), on the grounds that approximate measurement invariance by Bayesian Structural Equation Modelling is accepted (posterior predictive p-value =0.13; 95% confidence interval for difference between observed and replicated chi-square values (-11.35,44.36))

Residual correlations (male): acq4 with acq3, acq6 with acq1, acq2 with acq1
Residual correlations (female): acq4 with acq3; acq5 with acq4; acq6 with acq2

S15 Table. Model fit statistics for tests of factorial invariance of the ACQ6 (age)

Age (18-34, 35-54, ≥55 years)										
**Model**	**No. free parameters**	**Chi-square (df), p-value**	**AIC**	**BIC**	**SSA-BIC**	**RMSEA (90%CI)**	**p-close fit**	**CFI (ΔCFI‡)**	**TLI**	**SRMR**
**1.Configural invariance**	61	55.90 (20df), p<0.01	53040.96	56406.34	53212.52	0.04 (0.03,0.06)	0.80	1.00	0.99	0.01
**2. Weak measurement invariance (acq2-acq6) †**	51	97.51 (30df), p<0.01	53061.02	53366.51	53204.46	0.05 (0.04,0.06)	0.61	0.99 (-0.01)	0.99	0.03
**2b. Weak measurement invariance (acq1) †**	59	67.39 (22df), p<0.01	53048.97	53402.38	53211.91	0.05 (0.03,0.06)	0.69	1.00 (0.00)	0.99	0.02
**3. Strong measurement invariance**	41	212.89 (40df), p<0.01	53165.81	53411.39	53281.12	0.07 (0.06,0.08)	<0.01	0.98 (-0.02)	0.98	0.04
**3b. Partial measurement invariance ††**	45	117.25 (36df), p<0.01	53068.54	53338.09	53195.11	0.05 (0.04,0.06)	0.62	0.99 (-0.01)	0.99	0.03
**4. Factor means invariance †††**	43	170.68 (38df), p<0.01	53123.56	53381.13	53244.50	0.06 (0.05,0.07)	0.04	0.99 (-0.01)	0.98	0.07
**4b. Different latent mean for ages ≥55 years †††**	44	119.98 (37df), p<0.01	53069.20	53332.76	53192.95	0.05 (0.04,0.06)	0.64	0.99 (-0.01)	0.99	0.03

ACQ: Asthma Control Questionnaire; AIC: Akaike information criterion; BIC: Bayesian Information Criterion; CFI: Comparative Fit Index; df: degrees of freedom; RMSEA: Root Mean Square Error of Approximation ; SRMR: Standardised Root Mean Square Residual; SSA-BIC: Sample-Size Adjusted Bayesian Information Criterion; TLI: Tucker-Lewis Index

‡ relative to configural model
† items are assessed separately, with factor loading for acq1 fixed at 1 in model 2 and factor loading for acq2 fixed at 1 in model 2b
†† strong measurement invariance for acq2-acq5 only (not acq1 or acq6)
††† assuming partial measurement invariance (Model 3b) on the grounds that approximate measurement invariance by Bayesian Structural Equation Modelling is rejected (posterior predictive p-value <0.01; 95% confidence interval for difference between observed and replicated chi-square values (22.26,88.67))

Residual correlations (18-34 years): acq4 with acq3, acq6 with acq1
Residual correlations (35-54 years): acq4 with acq3, acq5 with acq4, acq6 with acq2
Residual correlations (≥55 years): acq4 with acq3, acq2 with acq1

S1 Fig. Sensitivity analysis: comparison of latent factor means and estimated means of the ACQ5 among severe asthmatic patients

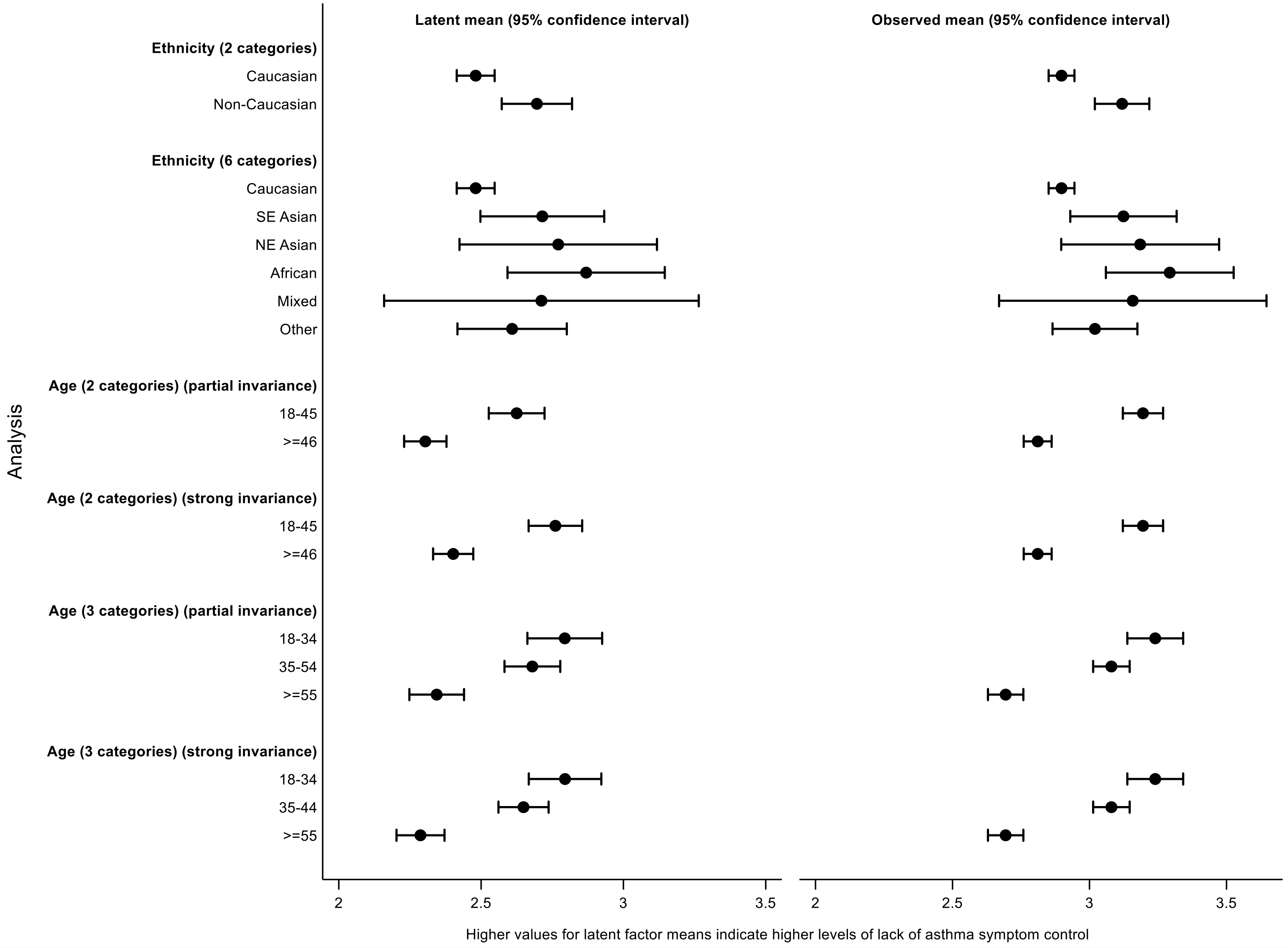

S2 Fig. Comparison of latent factor means and estimated means of the ACQ6 among severe asthmatic patients

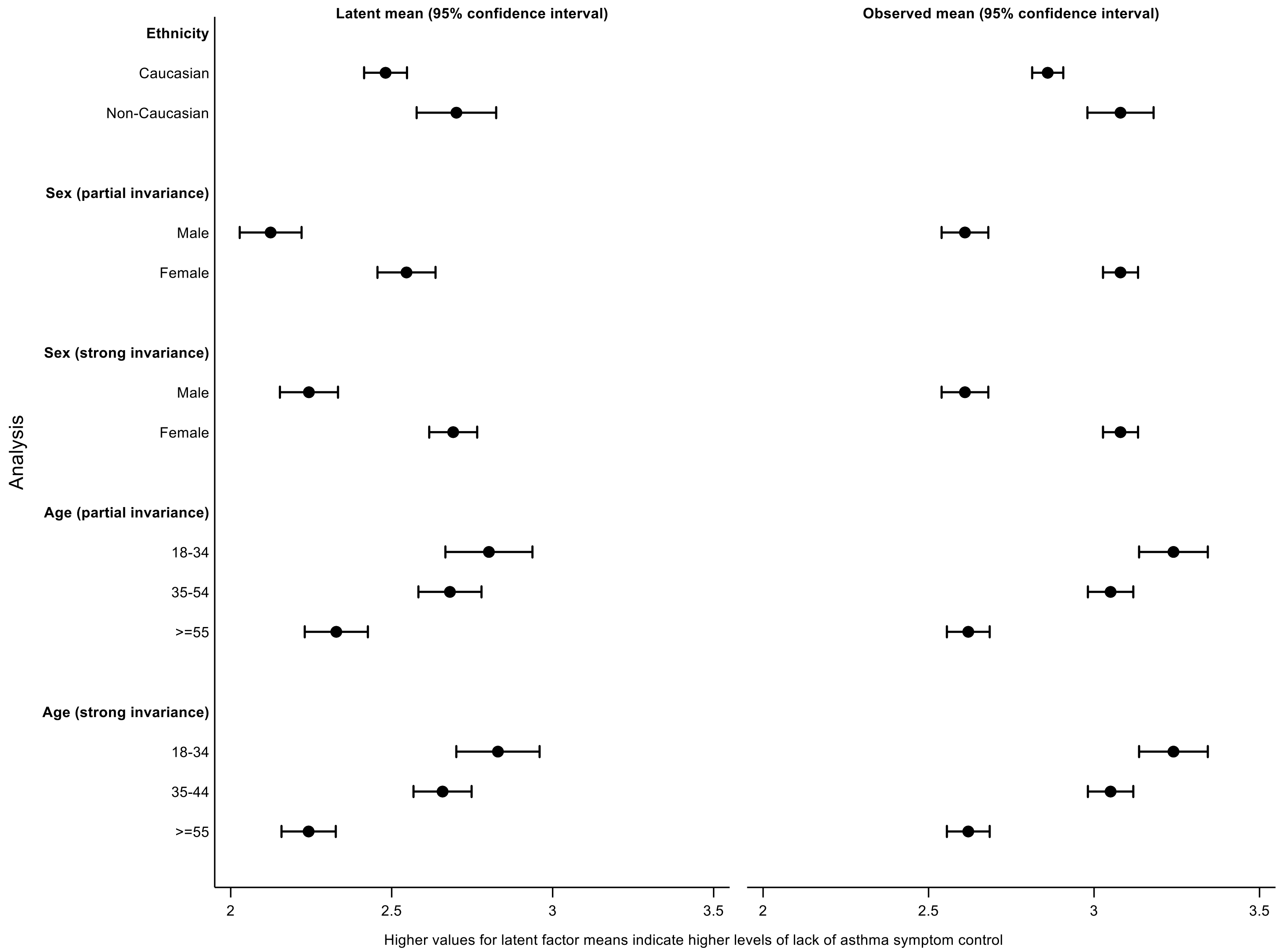

Supplement: S1 File — (PDF) [file pone.0295493.s001.pdf]
